# Reevaluating the 2.5 total-to-ionized calcium threshold during RCA-CKRT

**DOI:** 10.1186/s13054-026-06108-4

**Published:** 2026-05-29

**Authors:** Imran Khawaja, Naga Sumanth R. Gopireddy, Mony Fraer, Jonathan M. Nizar, Benjamin R. Griffin

**Affiliations:** 1 University of Iowa Hospitals and Clinics, 200 Hawkins Drive, Iowa City, IA 52242 USA; 2 Center for Access & Delivery Research and Evaluation (CADRE), Iowa City Veterans Affairs Health Care System, Iowa City, IA USA

We appreciate the thoughtful comments provided by Chardon et al. and welcome the opportunity to further clarify the aims of our work [1] and its contributions to the literature. In response to the question they pose, “are we diagnosing citrate accumulation or predicting mortality?” [2], we would argue that neither option truly reflects the clinical question we sought to address in our original article. Instead, our primary aim was to evaluate the clinical validity and utility of the widely used total-to-ionized calcium (tCa/iCa) threshold of 2.5 for guiding clinical decision-making and citrate dosing during continuous kidney replacement with regional citrate anticoagulation (RCA-CKRT).

Maintaining an uncorrected tCa/iCa ratio < 2.5 is a common recommendation in the RCA-CKRT literature [3], although the precise origins and physiologic justification for this cutpoint are difficult to determine. The earliest reference we identified was a case report from 1999 [4], followed by a larger retrospective analysis from the same group in 2001 demonstrating that a tCa/iCa ratio > 2.5 identified “citrate toxicity” in 12% of patients overall and in 33% of patients with liver failure, while also independently associating with mortality [5]. Although the explicit physiologic rationale for selecting 2.5 was not clearly established, this threshold subsequently became widely adopted throughout the RCA-CKRT literature. Our study was therefore designed primarily to evaluate the performance characteristics of this historically accepted threshold.

To that end, we examined the relationship between various tCa/iCa thresholds and mortality, using ROC analysis and related metrics to explore whether alternative thresholds might provide improved clinical discrimination. Chardon et al. suggest that the preferable approach would have been direct comparison with serum citrate levels or other directly measured laboratory markers [6]. However, we felt that methodology would not be able to address our clinical question. Serum citrate levels currently face the same conceptual limitations that tCa/iCa ratios do, as there is no well-established definition for citrate accumulation based on either serum levels or on clinical trajectories. It would therefore be difficult to evaluate the optimal tCa/iCa threshold based on these standards. Relatedly, we also note that the illustrative cases presented do not establish definitive misclassification, as without a gold standard their adjudication necessarily relies on indirect physiologic interpretation. In contrast, mortality represents an objective endpoint integrating the downstream physiologic consequences that motivate monitoring for citrate accumulation in the first place.

Chardon et al. try to draw a clear distinction between prediction of the serum citrate level and prediction of mortality, but we do not view these as easily separable questions in clinical practice. The reason clinicians monitor tCa/iCa during RCA-CKRT is based on the underlying assumption that impaired citrate metabolism and citrate accumulation lead to physiologic disturbances and downstream adverse outcomes. Within this conceptual framework, tCa/iCa is a surrogate marker for citrate accumulation, and citrate accumulation is itself a surrogate for harmful clinical effects. To the extent that tCa/iCa thresholds are used to guide citrate dosing and anticoagulation management, evaluation against an objective clinical outcome like mortality is appropriate and clinically relevant.

Our findings suggest that the conventional threshold of 2.5 fails to adequately identify patients at elevated clinical risk. Among those with an uncorrected tCa/iCa ratio < 2.5, albumin correction reclassified upward an additional 33 patients (Table 1). These reclassified patients demonstrated mortality rates nearly identical to patients exceeding the uncorrected 2.5 threshold (84.8% vs. 83.3%), and substantially higher than mortality among patients who remained below these thresholds (56.9%). While Chardon et al. propose that this may simply reflect additional prognostic information carried by albumin, we also show in Table 1 that lowering the threshold of the uncorrected tCa/iCa ratio to approximately 2.2 (without incorporating albumin information) produced reclassification patterns and mortality enrichment nearly identical to those observed using albumin-adjusted thresholds. These findings suggest that the improved performance associated with albumin correction primarily reflects recalibration of an empirically derived threshold rather than introduction of a new prognostic signal.

**Table 1:** Rates of reclassification and mortality outcomes with use of an albumin-corrected tCa/iCa threshold of 2.5 or an uncorrected threshold of 2.2, compared to the traditional uncorrected threshold of 2.5

Classification Group	Versus Corrected > 2.5		Versus Uncorrected > 2.2	
	*N*	Mortality	*N*	Mortality
Negative by Both	188	56.9%	180	55.6%
Reclassified Upward	33	84.8%	41	85.4%
Positive by Both	12	83.3%	12	83.3%

In this context, we do not view our findings as an attempt to redefine citrate accumulation or to develop a new mortality prediction tool. Rather, we show that the conventional tCa/iCa threshold of 2.5 inadequately identifies clinically relevant risk, and that either albumin adjustment or threshold recalibration with unadjusted ratios may improve the clinical utility of this widely used monitoring parameter. We agree that further work is needed to develop a validated, physiologically grounded definition of citrate accumulation, and we hope our findings contribute to that broader effort.

## Data Availability

The datasets used and/or analyzed during the current analysis are available from the corresponding author on reasonable request.

## Abbreviations

RCA: Regional citrate anticoagulation
CKRT: Continuous kidney replacement therapy
iCa: Ionized calcium
tCa/iCa: Total-to-ionized calcium ratio
